# The Coconut Water Antimicrobial Peptide CnAMP1 Is Taken up into Intestinal Cells but Does Not Alter P-Glycoprotein Expression and Activity

**DOI:** 10.1007/s11130-020-00826-y

**Published:** 2020-05-27

**Authors:** Katya Anaya, Maren Podszun, Octavio Luiz Franco, Carlos Alberto de Almeida Gadelha, Jan Frank

**Affiliations:** 1 Faculty of Health Sciences of Trairi, Federal University of Rio Grande do Norte, Santa Cruz, RN 59200-000 Brazil; 2 Institute of Nutritional Sciences, University of Hohenheim, D-70599 Stuttgart, Germany; 3 Department of Molecular Biology, Federal University of Paraíba, João Pessoa, PB 58051-900 Brazil; 4 Centro de Análises Proteômicas e Bioquímicas, Pós-Graduação em Ciências Genômicas e Biotecnologia, Universidade Católica de Brasília, Brasília, DF 70790-160 Brazil; 5 S-Inova Biotech, Pós-Graduação em Biotecnologia, Universidade Católica Dom Bosco, Campo Grande, MS 79117-900 Brazil

**Keywords:** Coconut antimicrobial peptide 1 (CnAMP1), Caco-2 cell line, LS180 cell line, Cellular uptake, P-glycoprotein activity

## Abstract

Coconut antimicrobial peptide-1 (CnAMP1) is a naturally occurring bioactive peptide from green coconut water (*Cocos nucifera* L.). Although biological activities have been reported, the physiological relevance of these reports remains elusive as it is unknown if CnAMP1 is taken up into intestinal cells. To address this open question, we investigated the cytotoxicity of CnAMP1 in intestinal cells and its cellular uptake into human intestinal cells. Considering the importance of the P-glycoprotein (P-gp) to the intestinal metabolism of xenobiotics, we also investigated the influence of CnAMP1 on P-gp activity and expression. Both cell lines showed intracellular fluorescence after incubation with fluorescein labelled CnAMP1, indicating cellular uptake of the intact or fragmented peptide. CnAMP1 (12.5–400 μmol/L) showed no signs of cytotoxicity in LS180 and differentiated Caco-2 cells and did not affect P-gp expression and activity. Further research is required to investigate the identity of CnAMP1 hydrolysis fragments and their potential biological activities.

## Introduction

Antimicrobial resistance was listed among the 10 threats to global health in 2019 by the World Health Organization [1], endangering the achievement of the Sustainable Development Goals. The discovery of new antibacterial molecules is a crucial step to overcome the challenge posed by the emergence of antibiotic resistance [2]. Antimicrobial peptides (AMP) are amino acid polymers with small sequence size, which present activity against microorganisms and are being considered a promising new class of antibiotics [3, 4].

Coconut water, the liquid endosperm from coconut (*Cocos nucifera* L.), has traditionally been used for medicinal purposes by ancient cultures and, more recently, several biologically active molecules were identified, including antifungal as well as antimicrobial peptides. Four peptides have been identified in green coconut water so far: a 10 kDa peptide with antifungal activity [5] and three peptides with antimicrobial activities (AMP), designated CnAMP1, CnAMP2 and CnAMP3 (composed of 9, 12 and 8 amino acids, respectively) [6]. CnAMP1 (≈ 860 Da) strongly inhibits the growth of fungi and Gram-positive and Gram-negative bacteria [7].

Food-derived bioactive peptides might exert health-beneficial effects by direct interaction with bacteria in the gut, by binding extracellular structures (*e.g*., plasma membrane receptors and transporters), or by being absorbed into intestinal cells, where they may interact with intracellular targets or be secreted and distributed to other tissues via the systemic circulation. Furthermore, peptides have been shown to be substrates and modulators of the activity of the P-glycoprotein (P-gp) efflux transporter [8]. P-gp is expressed on multiple barriers within the body, including the apical surface of intestinal cells [9] and plays an important role in the bioavailability of many drugs and phytochemicals. Changes in P-gp can increase or decrease the bioavailability of its substrates. The influence of Cn-AMP1 on P-glycoprotein would thus be an undesirable effect that raises safety concerns.

Thus, we investigated the cellular uptake of coconut water antimicrobial peptide CnAMP1 in LS180 and Caco-2 cells and its impact on cytotoxicity and P-glycoprotein activity.

## Material and Methods

### Material

Acetonitrile (ACN) and trifluoroacetic acid (TFA) were from J.T. Baker (Germany). NP-40 and 3-(4,5-dimethylthiazolyl)-2,5-diphenyl-tetrazoliumbromide (MTT) were from Sigma-Aldrich (Steinheim, Germany). Minimum Essential Medium (MEM), non-essential amino acid solution, pyruvate, fetal calf serum (FCS) and penicilin/streptomicin solution were obtained from Gibco (Germany). Dulbecco’s Modified Eagle Medium (DMEM) was purchased from Thermo Fisher Scientific (Germany). Bradford reagent Roti®-Quant was from Carl Roth (Germany). All the chemicals and reagents used were of HPLC or analytical grade.

Synthetic CnAMP1 peptide (SVAGRAQGM) and CnAMP1 labelled with fluorescein (5-FAM-SVAGRAQGM; Fluos-CnAMP1) were purchased from Peptide 2.0 Incorporated (Chantilly, VA, USA) and ProteoGenix (Schiltigheim, France), respectively. Purity of TCA-free peptide batches was >97%. Stock solutions (2 and 10 mmol/L) were prepared in sterile distilled water and stored at −20 °C.

### Cell Culture Conditions and Cell Differentiation

Caco-2 cells were cultivated in DMEM and LS180 cells in MEM. Both media were supplemented with 10% fetal calf serum, 100 IU/mL penicillin, 100 mg/mL streptomycin solution, 1% non-essential amino acids and 1% pyruvate. Caco-2 cells were maintained for 21 days after reaching total confluence for differentiation into small-intestinal-like cells and medium was renewed every two days (differentiation was confirmed by ZO-1 protein immunofluorescence). All cell lines were cultivated at 37 °C and 5% CO_2_.

### Cytotoxicity Assay

Cytotoxicity of CnAMP1 and Fluos-CnAMP1 against LS180 and Caco-2 cells was evaluated by MTT reduction assay [10]. Briefly, cells were seeded in 48-well plates at a density of 3 × 10^5^ cells (LS180) and 10^6^ cells (Caco-2) *per* well and incubated for 24 h at 37 °C, 5% CO_2_. Caco-2 cells were cultured following the protocol for differentiation described above. Supernatant was removed and cells received culture medium supplemented with CnAMP1 at different concentrations (12.5–400 μmol/L). Medium with 0.1% Triton X-100 was used as positive control and pure medium as negative control (100% viability). Treatments were randomized in the plate. After 48 h of incubation, 10 μL of the MTT solution [5 mg/mL in phosphate buffered saline (PBS)] was added to each well of the plate, which was placed in the incubator for 2 h. The blue formazan crystals were dissolved by the addition of 100 μL of solubilization reagent (99.4% DMSO, 0.6% acetic acid, 10% SDS). To dissolve the precipitate, the plates were then gently swirled for 5 min on a rotator shaker, at room temperature and protected from light. The absorbance was monitored at 580 nm (660 nm as background) using a Synergy HT microplate reader (BioTek Instruments GmbH, Bad Friedrichshall, Germany). Cytotoxicity was determined as a percentage of the maximum value after subtracting the background. Results were expressed as the percentage of each sample compared to the negative control and the assay was repeated three times with cells from different passages (between 45 and 50).

### Cellular Uptake of Fluos-CnAMP1

LS180 and Caco-2 cells were seeded on sterile coverslips and cultured as described above. LS180 cells were incubated until they reached 90–100% confluence. Caco-2 cells were used at the 21st day of differentiation. Media were removed and cells were washed twice with PBS prior the incubation. Before incubation with the peptide, cell DNA was stained with Hoechst. Fluos-CnAMP1 (100 μM prepared in glucose 1 g/L in PBS) was added to each well for 15, 30, 60 min or 24 h. Incubation was conducted in duplicates followed by the respective blanks (cells incubated only with PBS + glucose solution). After incubation, coverslips were washed five times with PBS and mounted on glass slides. Cells were not fixed in order to not alter membrane permeability. Slides were observed by fluorescence microscopy on a ZEISS Axiovert 100 M (Jena, Germany).

### P-Glycoprotein Expression *In Vitro*

Induction of P-gp was carried out as previously described by Abuznait and co-workers [11]. LS180 cells were seeded in 48-well plates at a density of 3 × 10^5^ cells *per* well and allowed to attach and grow to 50–60% confluence at 37 °C and 5% CO_2_. Different concentrations of CnAMP1 (12.5–200 μmol/L) were prepared in growth medium before use. Rifampicin, used as positive control, was prepared in DMSO and diluted to 25 μmol/L with medium. Incubation with CnAMP1 and controls were carried out for 48 h. LS180 cell lysates were prepared as follow: cells were washed with 200 μL PBS and 50 μL trypsin/EDTA solution were added to each well. Plates were incubated for 10 min at 37 °C, 5% CO_2_, after which 450 μL medium were added in order to inactivate the enzyme, and the cell suspension was then centrifuged for 5 min, 4 °C, 3000×*g*. Working on ice, cell pellets were resuspended in ice-cold PBS and centrifuged, 16,100×*g*; the supernatant was discarded and 20 μL of NP-40 lysis buffer with protease inhibitor cocktail added to the cell pellet. After 20 min of incubation on ice, cell pellets were sonicated for 30 s and centrifuged for 5 min, 4 °C, 16,100×*g*. The amount of protein in the supernatant was quantified according to the Bradford’s method. Supernatants were stored at −80 °C. The assay was repeated three times with cells in different passages (between 45 and 50) and P-gp expression determined by Western blotting.

### Western Blotting

Twenty-five micrograms of protein per lane were separated on a 7.5% SDS- polyacrylamide gel followed by transferring the proteins to a polyvinylidene difluoride membrane, which was blocked for 1 h (5% BSA in TBST) at room temperature. The primary antibody [P-gp, 1:5000 (Abcam); *β*-actin, 1:5000 (Santa Cruz Biotechnology)] was diluted with 5% BSA in TBST, and the membranes were incubated for 1 h at room temperature under gentle agitation. Membranes were washed and incubated with secondary antibody [goat anti-rabbit peroxidase conjugated, 1:10,000 (Calbiochem) diluted with 5% BSA in TBST] with gentle shaking for 1 h at room temperature. The bands were visualized using ImmunStar Western C Kit (Bio-Rad), and band intensity was recorded with a Peqlab Fusion Fx7 system (Vilber Lourmat, Eberhardzell, Germany). Relative concentrations of the proteins were quantified as the ratio of P-gp to *β*-actin band densities.

### P-Glycoprotein Activity Assay

The impact of CnAMP1 on P-glycoprotein (P-gp) activity was measured [11], using elacridar (3.5 μmol/L) as P-gp inhibitor and rifampicin as positive control (25 μmol/L). LS180 cells were seeded in 48-well plates at a density of 3 × 10^5^ cells *per* well and allowed to attach and grow to 50–60% confluence at 37 °C and 5% CO_2_. The fluorescent intensity of rhodamine 123 (Rh123) accumulated inside the cells was measured after 48 h using a Synergy HT microplate reader (Biotek, USA) under the excitation wavelength of 485 nm and emission wavelength of 529 nm and data acquisition was achieved using Gene5 software (Biotek). Data was normalized by the protein content. Cellular accumulation of Rh123 was determined as the fluorescent intensity per mg protein of each treatment sample in the presence of or absence of elacridar (P-gp inhibitor). Results were expressed as means ± standard deviation (SD) for cellular accumulation of Rh123 from treatment samples compared to control.

### Statistical Analysis

Data are presented as the mean ± SD. One-way analysis of variance (ANOVA) was performed for statistical comparison of the results, which was followed by Dunett’s test (in order to compare treatments with control) using GraphPad Prism (GraphPad Software Inc., San Diego, CA). Two-way analysis of variance was applied when necessary, followed by Bonferoni post-test. If *p* was <0.05, differences were considered statistically significant (*P < 0.05).

## Results and Discussion

### Cytotoxicity

**Fig. 1:**
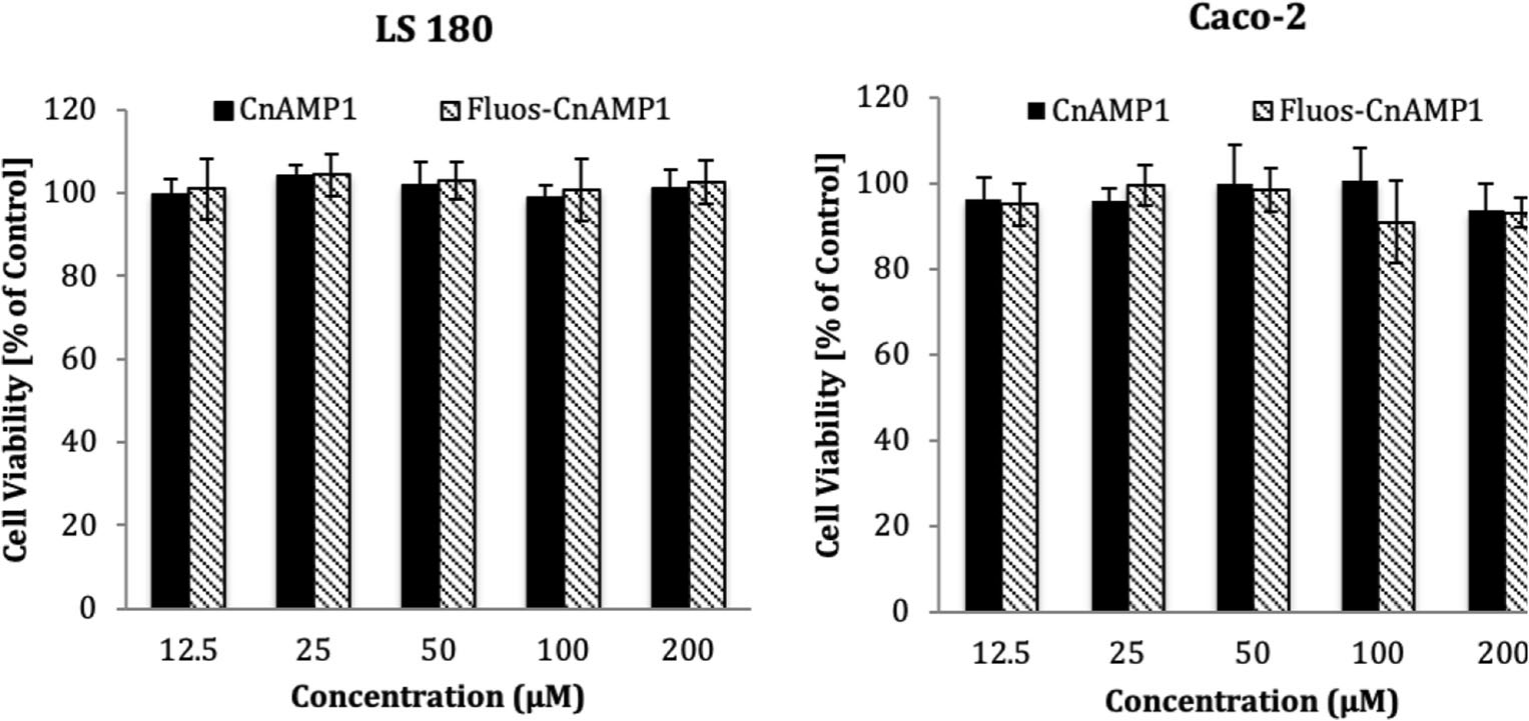
Viability of LS 180 and Caco-2 cells incubated with 12.5–200 μmol/L of CnAMP1 and Fluos-CnAMP1 for 48 h. Data represent mean ± SD of three independent experiments performed in triplicate. No statistical differences were observed

Cell viability assays revealed that CnAMP1 and Fluos-CnAMP1, for 48 h at concentrations up to 200 μmol/L, had no toxic effects on LS-180 and differentiated Caco-2 cells (Fig. 1). This is important given the regular coconut water intake in some regions of the globe or even considering the perspective of applying the peptide as a biopharmaceutical molecule. In agreement with our findings, Silva and co-workers [12] found no CnAMP1-induced cytotoxicity in murine macrophage-like cells (RAW 264.7). In non-differentiated Caco-2 cells, however, they observed that CnAMP1 reduced cell viability in a dose-dependent manner [12]. When grown for 21 days after confluence, Caco-2 cells differentiate into a small intestinal enterocyte-like phenotype [13]. The proteome of differentiated Caco-2 cells differs from undifferentiated cells in a number of proteins involved in cell recognition, structure, defence, transport, and signalling [14–16]. In particular, the increased expression of brush-border-associated hydrolases (such as aminopeptidase N and dipeptidases), membrane transporters and drug metabolizing enzymes in differentiated Caco-2 cells [15, 17] may explain the differences in susceptibility to CnAMP1-induced toxicity.

### Uptake of Fluos-CnAMP1 into Intestinal Cells

**Fig. 2:**
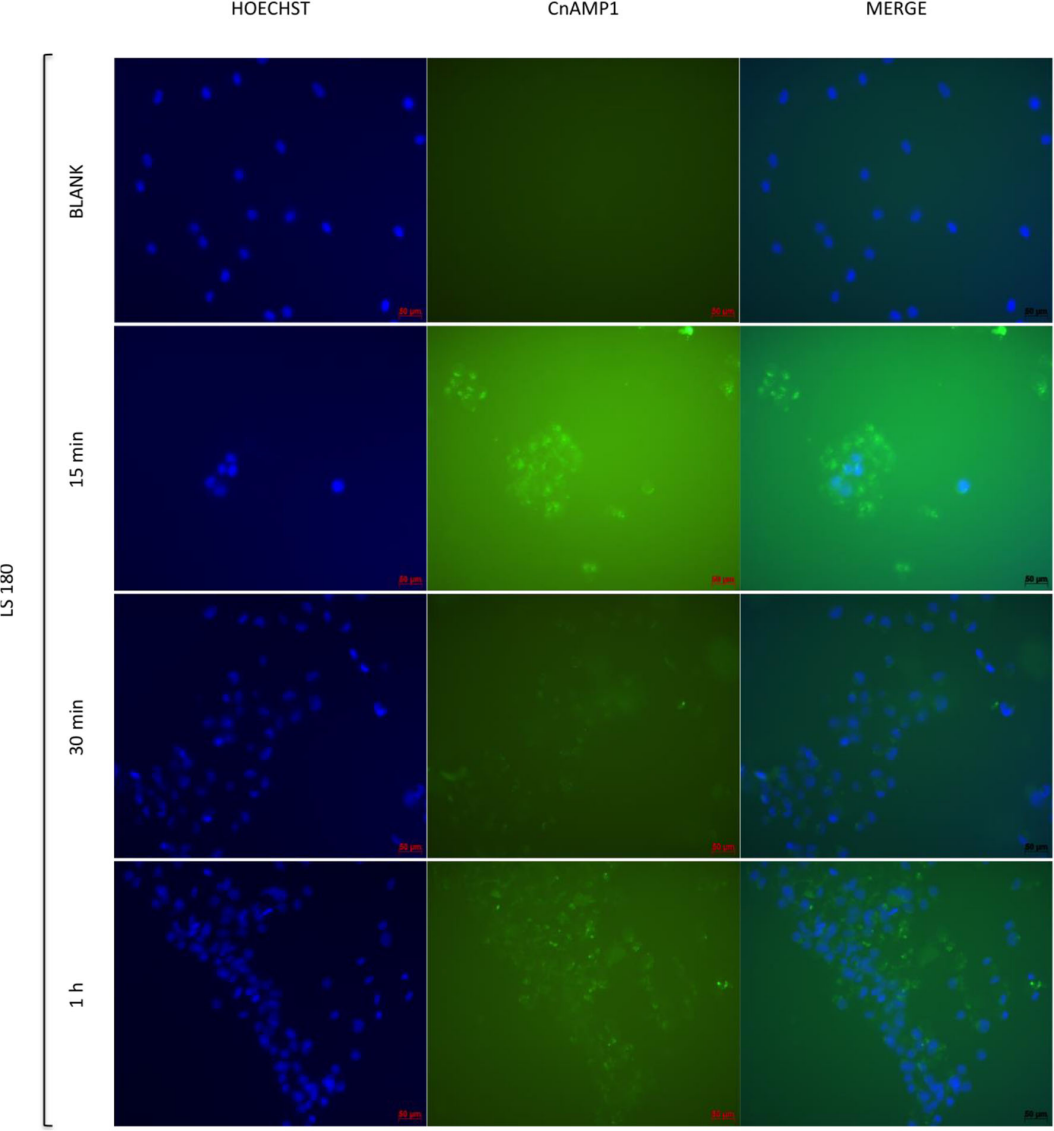
Indirect immunofluorescence microscopy of LS 180 cells incubated for 15, 30 or 60 min with 100 μmol/L fluorescence-labelled CnAMP1 (Fluos-CnAMP1). Blue: Hoechst DNA stain; green: Fluos-CnAMP1. Bars = 50 μm

**Fig. 3:**
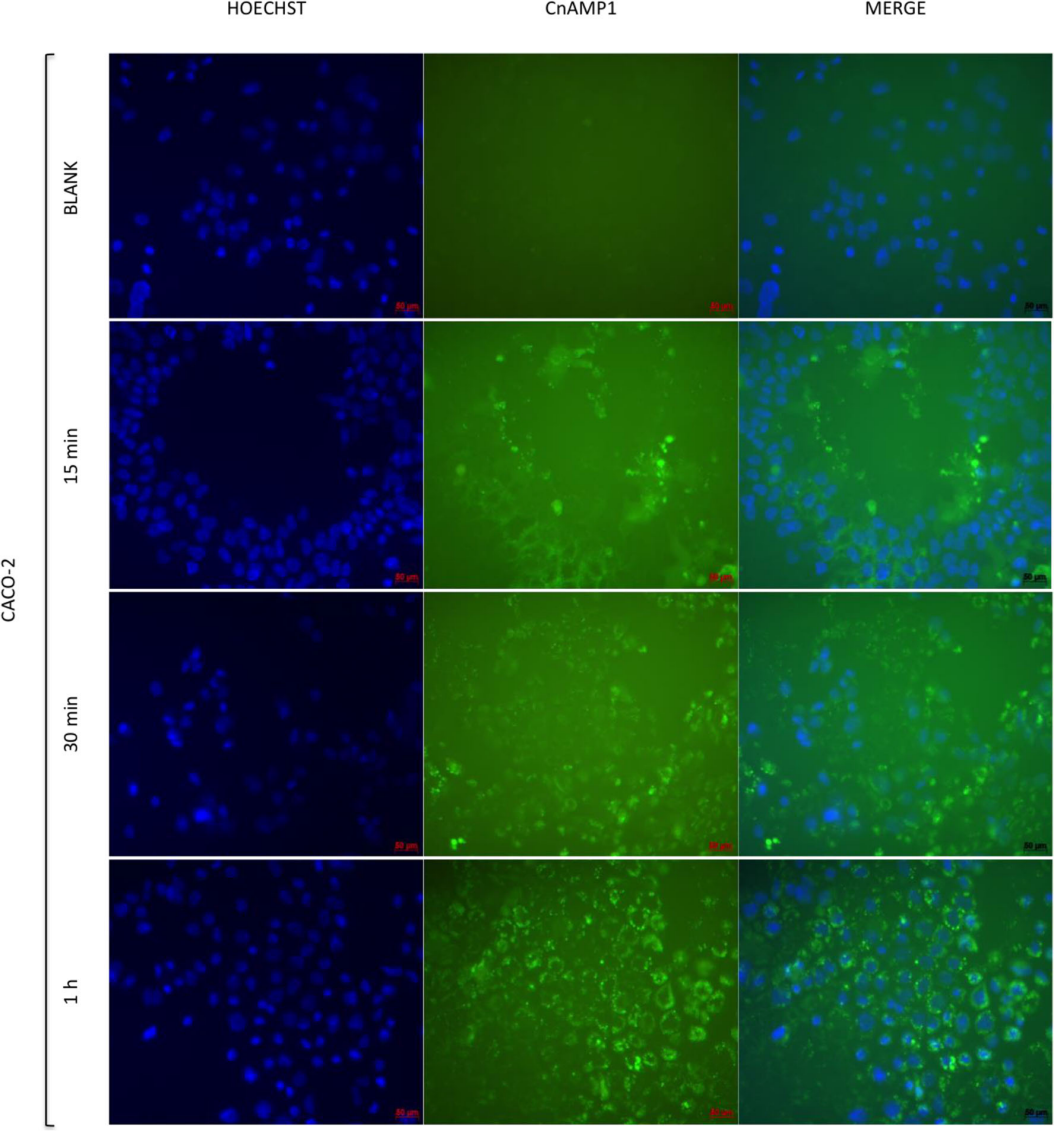
Indirect immunofluorescence microscopy of differentiated Caco-2 cells incubated for 15, 30 or 60 min with 100 μmol/L fluorescence-labelled CnAMP1 (Fluos-CnAMP1). Blue: Hoechst DNA stain; green: Fluos-CnAMP1. Bars = 50 μm

In our previous study, we observed a gradual decline of non-labelled CnAMP1 in cell culture supernatant but no CnAMP1 within the cell pellet, suggesting extensive hydrolysis by brush border peptidases [18]. To investigate this hypothesis and examine if the cells are capable to absorb CnAMP1 or its breakdown fragments, LS180 and Caco-2 cells were incubated with fluorescein-labelled CnAMP1. After 15 min, a fluorescence signal emanated by the cells was already detected (Figs. 2 and 3). At that time point, it was not possible to distinguish if the fluorescent peptide was bound extracellularly to the membrane or internalized. After 1 h of incubation, the fluorescence signal was stronger in Caco-2 than in LS180 cells. These results are in line with the rapid disappearance of CnAMP1 from the Caco-2 supernatant, in comparison with LS180 cell, when non-labelled peptide was incubated with these two cell lines in our previous experiments. Micrographs of cells after 1 h incubation with Fluos-CnAMP1, especially those from Caco-2 cells, show fluorescent vesicles localized in the cytosol. Therefore, the addition of the fluorescent-label either inhibited hydrolysis of CnAMP1 by brush border peptidases, which could thus be internalised in its native form, or a hydrolysis product, carrying the label, was taken up into LS180 and Caco-2 cells. Incubation for 24 h resulted in complete cell detachment and no cells remained on the coverslip after the washing cycles, which precluded the recording of the 24 h micrographs.

Interestingly, another study also observed that the hydrolysis product of a casein-derived peptide (VLPVPQK) was absorbed by Caco-2 cells and secreted into the basolateral chamber in a trans-well assay, indicating that the hydrolysis product might reach the systemic circulation *in vivo* [19]. Similar findings were reported for another milk-derived peptide (LHLPLP), for which partial hydrolysis by Caco-2 brush border peptidases were observed prior to the flux across the cell layer. The LHLPLP hydrolysis product had a higher flux than its precursor and was proposed to be responsible for the antihypertensive effects of LHLPLP in animal models [20].

Gastrointestinal enzymatic breakdown of dietary bioactive peptides is a recurring concern, as hydrolysis could, potentially, inactivate them. However, after simulated gastrointestinal digestion, peptide fragments can preserve their original biological activities [21–23]. Moreover, the smaller the peptide (di- and tri-peptides) the higher its chance to be more efficiently internalized by intestinal cells [24]. Before transported into the bloodstream, bioactive sequences could also play new and important roles in regulation of nutrient absorption and modulation of epithelial cell functions [25]. The activity of intact CnAMP1 or its fragments could be relevant independently of their brush border uptake. They could, at the level of the gut lumen, either modify the intestinal microbiota (as reported for the duck egg white-derived peptide VSEE [26]) or interact with receptors on the surface of the intestinal epithelium.

Human trials have demonstrated that small food-derived peptides resistant to exopeptidases can remain bioavailable, circulate in the blood and reach target organs [27–30]. Therefore, further studies are necessary to completely understand the hydrolysis of CnAMP1 at the intestinal barrier, the absorption of the hydrolysis products into the cells, their secretion at the basolateral membrane, their possible efflux to the gut lumen as well as their potential biological activities.

### P-Glycoprotein Expression and Activity

**Fig. 4:**
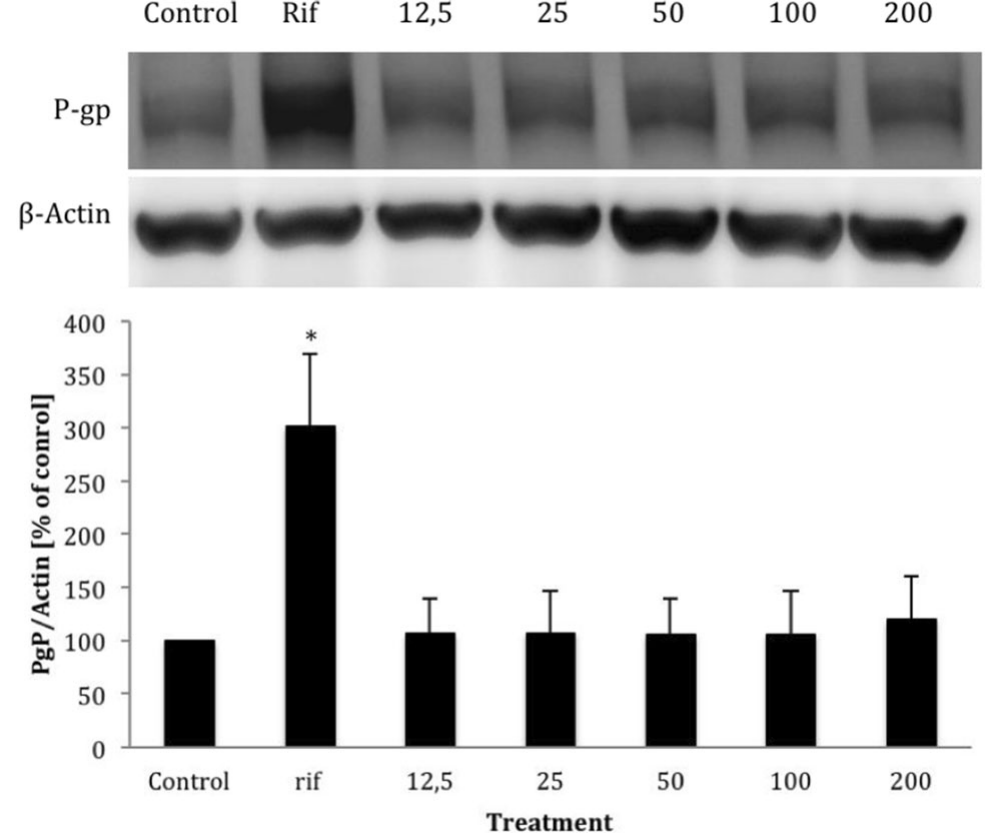
Representative Western blot and band densities of P-glycoprotein (P-gp) protein expression (standardised to *β*-actin) in LS180 cells incubated for 48 h with increasing concentrations of CnAMP1. Rifampicin (25 μmol/L) was used as positive control. Data represent mean ± SD of three independent experiments performed in triplicate. *Significantly different from control (*p* < 0.05, one-way ANOVA)

**Fig. 5:**
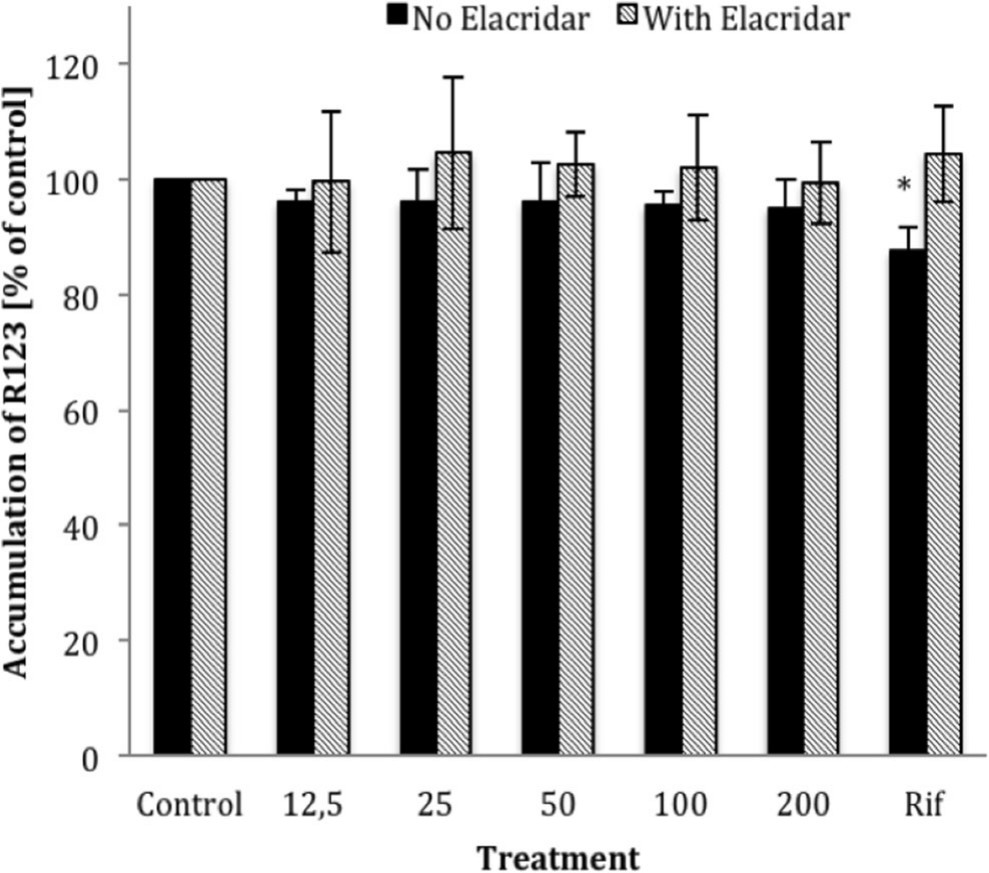
P-glycoprotein (P-gp) activity (intracellular accumulation of the P-gp substrate rhodamine 123) quantified in LS-180 cells incubated with 12.5–200 μmol/L CnAMP1 for 48 h. P-gp activity was determined in the presence (black bars) or absence (dashed bars) of the P-gp inhibitor Elacridar. Data represent mean ± SD of three independent experiments performed in triplicate. *Significantly different from control (one-way ANOVA) and significantly different from cells in the presence of Elacridar (two-way ANOVA) at *p* < 0.05

For safety considerations, it is important to know whether a newly identified natural compound is an inhibitor or inducer of P-gp and may potentially alter the bioavailability of concurrent ingested compounds. We therefore investigated if CnAMP1 inhibits or induces the expression and transport activity of P-gp. However, neither the expression (Fig. 4), nor the activity of the membrane transporter (Fig. 5) was altered in LS180 cells upon incubation with 12.5–200 μmol/L CnAMP1 for 48 h. P-glycoprotein (P-gp) is an efflux transporter expressed in the plasma membrane of epithelial cells, including those of the intestine, which shuttles xenobiotics out of the cell. P-gp is thus an important part of the cellular defence against potentially harmful substances. P-gp has a broad substrate affinity and transports a vast array of chemically and structurally unrelated compounds [31, 32]. P-gp activity in intestinal epithelial cells greatly influences the bioavailability of many compounds and, thus, P-gp is often involved in drug interactions affecting the pharmacokinetics and pharmacodynamics of drugs, which may ultimately alter their safety and efficacy [9]. Our results suggest that CnAMP1 does not elicit unfavourable drug interactions.

## Conclusions

In summary, we gathered evidence that intestinal epithelial cells may internalize CnAMP1 brush-border hydrolysis products. Additional experiments are required to identify all hydrolysis products and their uptake kinetics in intestinal cells. Future research should also focus on whether the small fragments of CnAMP1 maintain the previous reported activities or display novel systemic biological effects. We demonstrate here that CnAMP1 did not interfere on P-gp expression and activity, consequently it does not modify the bioavailability of xenobiotics. These results, along with the absence of cell toxicity, reinforce the safety aspects related to coconut water consumption as well as the use of CnAMP1 as potential novel antimicrobial compound.

## Abbreviations

CnAMP1: coconut antimicrobial peptide-1
FCS: fetal calf serum
MEM: minimum essential medium
MTT: 3-(4,5-dimethylthiazol-2-yl)-2,5-diphenyltetrazolium bromide
P-gp: P-glycoprotein
Rh123: rhodamine 123
TBST: Tris buffered saline with Tween 20
TCA: trichloroacetic acid
